# Chemotherapy-induced acute kidney injury: epidemiology, pathophysiology, and therapeutic approaches

**DOI:** 10.3389/fneph.2024.1436896

**Published:** 2024-08-09

**Authors:** Rafaella Maria da Cunha Lyrio, Bruna Reis Araújo Rocha, Ana Luiza Rodrigues Mascarenhas Corrêa, Maria Gabriela Santana Mascarenhas, Felipe Luz Santos, Rafael da Hora Maia, Lívia Benezath Segundo, Paulo André Abreu de Almeida, Clara Magalhães Oliveira Moreira, Rafael Hennemann Sassi

**Affiliations:** ^1^ Department of Medicine, Universidade Salvador (UNIFACS), Salvador, Brazil; ^2^ Hematology Department, Hospital de Clínicas de Porto Alegre (HCPA), Porto Alegre, Brazil

**Keywords:** acute kidney injury, conventional chemotherapy, nephrotoxicity, cancer, onconephrology

## Abstract

Despite significant advancements in oncology, conventional chemotherapy remains the primary treatment for diverse malignancies. Acute kidney injury (AKI) stands out as one of the most prevalent and severe adverse effects associated with these cytotoxic agents. While platinum compounds are well-known for their nephrotoxic potential, other drugs including antimetabolites, alkylating agents, and antitumor antibiotics are also associated. The onset of AKI poses substantial risks, including heightened morbidity and mortality rates, prolonged hospital stays, treatment interruptions, and the need for renal replacement therapy, all of which impede optimal patient care. Various proactive measures, such as aggressive hydration and diuresis, have been identified as potential strategies to mitigate AKI; however, preventing its occurrence during chemotherapy remains challenging. Additionally, several factors, including intravascular volume depletion, sepsis, exposure to other nephrotoxic agents, tumor lysis syndrome, and direct damage from cancer’s pathophysiology, frequently contribute to or exacerbate kidney injury. This article aims to comprehensively review the epidemiology, mechanisms of injury, diagnosis, treatment options, and prevention strategies for AKI induced by conventional chemotherapy.

## 1 Introduction

Conventional chemotherapy remains the main anti-cancer therapy for several patients in spite of all the new advancements in this area. Its use, however, is frequently limited by the many adverse effects associated with this type of therapy. Acute kidney injury (AKI), characterized by a sudden decline in renal function, stands as a critical complication of chemotherapy, jeopardizing patient well-being, survival, and treatment effectiveness.

According to the Kidney Disease Improving Global Outcomes (KDIGO) criteria, AKI is defined as: a ≥0.3 mg/dL increase in serum creatinine (sCr) within 48 hours; a 1.5-fold increase in baseline creatinine within 7 days; or a urinary output of less than 0.5 ml/kg/h within 48 hours. Although universally applied nowadays, some older studies may have used different parameters, leading to a highly variable incidence of AKI in the literature.

The development of chemotherapy-induced AKI is extremely detrimental to patient’s health, with chemotherapy discontinuation or dose reduction frequently necessary and contributing to decreased treatment efficacy and overall survival. This complication results in an in-hospital mortality rate of 15% and a 48% chance of renal function non-recovery (1). The frequency and severity are largely dependent on the specific chemotherapy drug being used. Cisplatin, for instance, accounts for 20% of all chemotherapy-related AKI episodes in hospitalized cancer patients (2), with lower, but significant rates associated with antimetabolites, antitumor antibiotics, and alkylating agents.

The pathophysiology of chemotherapy-induced AKI is multifaceted and not completely understood in most cases, involving a complex interplay of direct nephrotoxic effects, altered systemic hemodynamics, and immune-mediated responses. Since conventional chemotherapy does not specifically target malignant cells, it often causes collateral damage to the delicate renal microenvironment, precipitating oxidative stress, inflammation, and renal tubular injury. It is noteworthy that the mechanism of renal injury varies based on the chemotherapy agent, with acute tubular injury (ATI) as the main cause of AKI in conventional chemotherapy (3–5) (**Table 1**). Thrombotic microangiopathy (TMA) proved to be more common among antimetabolites and antitumor antibiotics, while interstitial nephritis is mainly seen in Oxaliplatin (6–8). Vasoconstriction, altered intraglomerular hemodynamics, and crystal deposition are observed in some studied drugs (9–11).

**Table 1:** Anticancer drugs and histopathology of AKI.

Pharmacological class	Drug	Renal histopathology
Platinum compounds	Cisplatin Carboplatin Oxaliplatin	ATI ATI, AIN ATI, AIN, TMA
Antimetabolites	Methotrexate Pemetrexed Clofarabine Gemcitabine Capecitabine Cytarabine	ATI, Crystal nephropathy ATI ATI TMA TMA ATI, Crystal nephropathy
Antitumor antibiotics	Mitomycin C Doxorubicin	TMA ATI
Alkylating agents	Ifosfamide Trabectedin Bendamustine Streptozocin Melphalan	ATI Myoglobin-induced tubular damage ATI ATI ATI

ATI, acute tubular injury; AIN, acute interstitial nephritis; TMA, thrombotic microangiopathy.

Individual susceptibility to chemotherapy-induced AKI is influenced by a myriad of factors, with advanced age and a higher drug concentration over time being the most frequently associated (12–15). As expected, the presence of comorbid conditions, especially a prior history of renal dysfunction stood out as an important predictor of AKI (16, 17). Some drugs showed variable metabolism and transport among individuals, possibly due to genetic variation, ultimately leading to a higher occurrence of AKI (18, 19). Lastly, the concomitant administration of a second nephrotoxic agent or diuretic may also predispose (20, 21).

While the recognition of chemotherapy-induced AKI as a significant clinical entity continues to grow, its prevention remains challenging. A common approach is using intravenous (IV) hydration with isotonic saline to maintain adequate urine output and volemic status aiming to avert renal damage. More tailored interventions may be employed based on the specific chemotherapy regimen, such as sodium bicarbonate infusion and Leucovorin rescue for patients receiving methotrexate or pemetrexed (22). Although various preventive strategies have been explored, their real-world application is limited, including Protein A immunoadsorption in patients receiving mitomycin C (23). Numerous of these measures, such as the mannitol use in patients treated with cisplatin, remain controversial (24, 25). Further research is warranted to better inform therapeutic approaches in this domain.

Despite the high frequency of chemotherapy-induced AKI, there are few therapeutic options once the damage has been established. Most interventions are only supportive including electrolyte management and dialysis when needed. Newer biomarkers are being researched to detect AKI in a subclinical setting and prompt timely intervention (26).

In this review article, we explore AKI induced by conventional chemotherapy, delving into the pathophysiological mechanisms, risk factors predisposing to renal injury, diagnostic strategies guiding early recognition, and therapeutic interventions aimed at mitigating nephrotoxicity and preserving renal function. We endeavor to empower clinicians with the knowledge and tools necessary to optimize renal health in the oncology setting and enhance the therapeutic index of chemotherapy regimens.

## 2 Article type

This is a review article discussing AKI induced by conventional chemotherapy.

## 3 AKI-associated chemotherapeutic drugs

### 3.1 Platinum compounds

#### 3.1.1 Cisplatin

Cisplatin is an inorganic platinum compound widely used as a chemotherapy drug to treat various types of solid tumors, including lung, genitourinary, head, and neck (27). Its main mechanism of action revolves around its ability to crosslink with the purine bases on the DNA, thereby inhibiting cell replication and promoting apoptosis (28).

The nephrotoxicity of cisplatin has been well recognized since its introduction more than four decades ago (29). AKI is a common and major side effect affecting about one third of patients exposed to the drug thus limiting the dose that can be safely administered (30). Incidence rates may vary according to different criteria for diagnosis of AKI, with recent studies reporting even higher percentages, ranging from 53.7% (RIFLE) to 69% (KDIGO) (31, 32). The majority of cases, comprising 83%, manifest in stage 1 AKI according to KDIGO criteria, with only 5% presenting at stage 3 (32). The etiological pathways underlying Cisplatin-induced acute kidney injury (CIA) remain incompletely elucidated but are predominantly attributed to the generation of reactive oxygen species (ROS) and disruption of antioxidant defense mechanisms, such as the depletion of intracellular glutathione (GSH) in renal tubular cells (33, 34). Concurrently, an inflammatory cascade, characterized by heightened tumor necrosis factor alpha (TNF-α) levels alongside other cytokines, exacerbates tubular injury (35, 36). This cascading effect precipitates degenerative, apoptotic, and necrotic processes within the epithelium of renal tubules, particularly prominent in proximal convoluted tubules due to their elevated platinum concentration, as evidenced by renal pathology (3, 37–39). Concomitant renal vasoconstriction, implicated in reducing renal blood flow, further exacerbates renal compromise (9). Its deleterious effects on the kidneys are dose, duration, and frequency dependent. Higher doses (e.g. above 100 mg/m2), cumulative doses, and an elevated peak platinum concentration in plasma above 6µg/ml are associated with elevated rates of kidney injury (30, 40, 41). Retrospective studies suggest that a weekly infusion of cisplatin at reduced doses may be an alternative to the high-dose 3-weekly infusion, resulting in a lower incidence of AKI while maintaining similar progression-free survival rates (31).

AKI during cisplatin administration is influenced by various factors, with advanced age emerging as a risk factor, as suggested by multiple studies (30, 42, 43). Furthermore, female gender, smoking, and hypoalbuminemia have been associated with increased risk (43). The presence of hypertension, chemotherapy-induced nausea and vomiting, cardiovascular disease, diabetes mellitus, and advanced cancer further heightens the likelihood of AKI (32, 44, 45). Ethnicity may also be a contributing factor, with African American individuals at possibly higher risk (46). A comprehensive risk assessment tool integrating nine factors, including age, hypertension, diabetes mellitus, smoking status, hemoglobin level, white blood cell count, serum albumin level, serum magnesium level, and cisplatin dose offers a valuable predictive mechanism (47). Additionally, the genetic factor plays a role, with carriers of the gene polymorphism OCT2/SLC22A2 experiencing less nephrotoxicity. This finding is attributed to organic cation transporter 2 (OCT2), which is implicated in the cellular uptake of cisplatin (18).

Pantoprazole has shown promise in reducing CIA by inhibiting OCT2. Human studies indicate its effectiveness and animal studies support its potential through mechanisms such as reducing inflammation, oxidative stress, and apoptosis in renal cells induced by cisplatin (48–50). The recommended dose of Pantoprazole is 40 mg intravenously, administered concurrently with cisplatin (48). However, this protective effect of Pantoprazole was not observed in children with Osteosarcoma treated with a combination of Cisplatin, Methotrexate, and Doxorubicin (51). Other inhibitors of OCT2, such as Lansoprazole and Imatinib, have also been reported to reduce CIA in animal models (52, 53). Additionally, by the same mechanism, Cimetidine has shown protective effects in both animal and human studies, whether used alone or in combination with verapamil, and without interfering with the antitumor effects of cisplatin (54–58).

Given the severity of AKI, the dose of cisplatin should be kept at the minimum necessary and efforts made to avoid concomitant use of nephrotoxic agents. Several potential preventive measures for CIA have been explored with variable results. The most widely used is IV hydration with isotonic saline, which increases renal blood flow and induces diuresis without affecting the antitumor effect (59). The recommendation of isotonic saline by the FDA is 1-2 liters infused for 8 to 12 hours before cisplatin injection (60). However the optimal dose remains unclear. A systematic review comparing hydration methods for cisplatin treatment found that a shorter, lower-volume hydration regimen (1.9 to 4.3 liters over 4-5 hours) resulted in less kidney damage compared to the conventional approach (4.5 to 7.8 liters over 24 hours or more) (61). Recent studies have also proposed oral hydration solutions after cisplatin infusion as an alternative to IV hydration in preventing AKI among outpatients, but concerns regarding anorexia have led to a lack of recommendation (62–64).

Hypomagnesemia, a common side effect of cisplatin affecting up to 90% of patients, has been associated with exacerbating nephrotoxicity (65–67). Magnesium supplementation has emerged as a promising preventive measure, supported by several studies. The exact protective mechanism remains incompletely understood, with animal models suggesting that hypomagnesemia may heighten inflammatory response, dehydration, and upregulation of OCT2, leading to increased renal accumulation of cisplatin and worsening of CIA (68, 69). Meta-analyses advocate for incorporating 1–3 g of IV magnesium in the hydration protocol during cisplatin infusion, emphasizing its cost-effectiveness and potential efficacy in preventing CIA while maintaining antitumor efficacy (70, 71). Other studies further reinforce the protective effect of IV magnesium as a premedication, independent of serum magnesium levels and the risk of CIA (72–74). Despite the overall positive outcomes reported, uncertainties persist regarding the optimal dosage of magnesium supplementation before and after administration (75).

The use of diuretics for preventing CIA remains controversial. Mannitol, an osmotic diuretic not absorbed by renal tubules, has been associated with a lower increase in sCr levels, particularly in high cisplatin doses (>= 100 mg/m2), and patients with hypertension (25, 76, 77). On the other hand, concerns about hyponatremia and overdiuresis have been raised, and studies did not show a reduction in the risk of AKI with mannitol compared with saline hydration alone (24). Moreover, hydration alone or in combination with furosemide may result in less kidney injury when compared to saline and mannitol combination, but without evidence supporting the superiority of furosemide over hydration alone (78).

Amifostine (ETHYOL) has been shown to decrease nephrotoxicity in preliminary clinical studies (79). In humans a phase III trial in women with ovarian cancer found that amifostine reduced the incidence of nephrotoxicity from 33 to 10 percent with six cycles of a chemotherapy regimen that included cisplatin (100 mg/m2) (80). Amifostine is currently approved by the FDA to reduce the cumulative renal toxicity associated with repeated administration of cisplatin in patients with ovarian cancer. However, it may be limited due to adverse effects (e.g. hypotension, nausea, vomiting) and the elevated cost (81). Although there are concerns about possible reduction of antitumor efficacy, the data so far has shown no decrease in effectiveness against tumors, as evaluated through histopathological examination (80). Its mechanism involves free radical scavenging, DNA protection, and induction of cellular hypoxia (82). The recommended FDA dose is 910 mg/m2, IV, once daily, starting 30 minutes before chemotherapy administration and infused over 15 minutes (83).

Diethyldithiocarbamate (Immuthiol), a platinum chelator, has its usage restricted due to its ototoxic effects (84). Similarly, GSH, despite its antioxidant properties that can interact with electrophilic compounds to reduce cell damage and potentially mitigate CIA, is limited due to neurotoxicity (85, 86). Sodium thiosulfate, an inorganic compound, has shown limited benefit as it decreases the antitumor efficacy of cisplatin and only minimally reduces nephrotoxicity (87). Fosfomycin also did not demonstrate significant benefits (88).

Various animal and human studies have demonstrated that several natural products and antioxidants, including Resveratrol, Quercetin, Lycopene, Melatonin, Capsaicin, vitamins C and E, Selenium, Glutamine, and N-acetylcysteine (NAC) are potentially effective in reducing CIA due to their anti-inflammatory properties, ability to reduce oxidative stress, and balancing of antioxidant enzymes (89–105). Furthermore, IL-10, TNF-alpha inhibitors such as salicylates, and the PPAR agonists fibrate and rosiglitazone, have shown promise against cisplatin nephrotoxicity through their anti-inflammatory effects (106–109). Anti-apoptotic agents such as inhibitors of p53, mitogen-activated protein kinase (MAPK), and c-Jun N-terminal kinase (JNK), along with cell cycle inhibitors and interferences in the metabolization of cisplatin may play a role in mitigating CIA (110–113).

In severe cases of CIA, temporary hemodialysis may be required. Although renal function often shows signs of reversibility and nonprogression, a significant proportion of patients may experience a slight to moderate long-term decline in renal function (30, 32, 114, 115). However, no instances of End-Stage Renal Disease (ESRD) necessitating permanent dialysis have been reported (30, 31). The severity of long-term renal impairment is closely associated with the cumulative cisplatin dose administered (40). Premature discontinuation of high-dose cisplatin therapy is observed in approximately 17%-33% of patients experiencing CIA, making it the major factor for interruption of cisplatin treatment (32, 116).

The diagnosis of CIA traditionally relies on SCr and blood urea nitrogen (BUN) measurements. However, these markers may not reflect minor decreases in glomerular filtration rate (GFR) until significant impairment has occurred (117). Research is thus exploring new biomarkers for early CIA detection before changes in traditional markers become apparent. IL-18 demonstrates increased urine excretion post tubular injury but it has several confounding factors, limiting its use (26, 118, 119). Kidney injury molecule 1 (KIM-1), an FDA-approved urinary biomarker for drug-induced proximal tubular injury, has high sensitivity and specificity for detecting CIA, which typically raises within 3 days (118, 120). Combining KIM-1 with monocyte chemotactic protein-1 (MCP-1) enhances early detection (121). Neutrophil gelatinase-associated lipocalin (NGAL), released during renal injury, can be identified as early as three hours post-cisplatin infusion, with higher levels after twelve hours, showing effectiveness in early CIA detection compared to traditional markers, cystatin C, and albuminuria (26, 119, 122, 123). The NGAL-creatinine ratio improves sensitivity and specificity within the first 24 hours (124). KIM-1 and NGAL are more sensitive than SCr, which rises only 3-6 days after cisplatin administration (125). Their transient elevation, up to 7 days, may predict AKI risk in subsequent cisplatin cycles. Other biomarkers such as fatty acid-binding protein (L-FABP) and leucine aminopeptidase (LAP) also show promise (26). Individual variation necessitates baseline assessments before treatment initiation. These non-invasive biomarkers enable risk stratification, detecting subclinical kidney damage and facilitating prompt intervention. Integrating them with traditional assays could improve clinical outcomes in cisplatin-treated patients, although further studies are needed to establish appropriate cut-off values for early CIA detection (126).

#### 3.1.2 Carboplatin

Carboplatin, a second-generation platinum-based chemotherapy agent introduced in 1981, is commonly used in the treatment of ovarian, germ cell, and head and neck cancers (127, 128). Compared to cisplatin, carboplatin is associated with a markedly reduced incidence of adverse effects, notably a lower risk of renal dysfunction. This reduced nephrotoxicity is largely attributed to the substitution of chloride with a bidentate cyclobutane dicarboxylate in the cis position of its molecular structure (129).

Despite its improved safety profile, carboplatin can still induce mild forms of AKI. Most cases are characterized by a reduction in creatinine clearance (CrCl) by 25% to 50%, typically observed following subsequent courses of carboplatin treatment (130–132). Additionally, increased urinary markers of tubular damage, such as amine aminopeptidase (AAP) and N-acetyl-β-D-glucosaminidase (NAG), have been detected from the fourth cycle of treatment onwards (132). Case reports have linked carboplatin regimens to instances of focal tubular necrosis and acute interstitial nephritis (AIN), as confirmed by renal biopsies, and exhibited significant renal function improvement following oral prednisone therapy at a dose of 1 mg/kg/day for four weeks. In one such case, the patient required temporary hemodialysis (131).

Prior exposure to cisplatin is a notable risk factor for renal injury during subsequent carboplatin administration (131). Moreover, the nephrotoxicity associated with carboplatin is dose-dependent, with doses exceeding 800 mg/m² of body surface area being linked to AKI after the initial cycle of chemotherapy (133).

To mitigate the risk of carboplatin-induced AKI, pre-hydration with IV isotonic saline is commonly employed, especially in patients receiving high doses ranging from 800 mg/m² to 1,600 mg/m² (59). The FDA has recommended a dose reduction protocol based on baseline CrCl: for patients with a CrCl of 41 to 59 mL/min, the advised dose is 250 mg/m², while for those with a CrCl of 16 to 40 mL/min, a dose of 200 mg/m² is recommended (134).

#### 3.1.3 Oxaliplatin

Oxaliplatin, a third-generation platinum compound, is utilized in the treatment of various cancers, notably colorectal cancer (135). This chemotherapeutic agent is conjugated to a 1,2-diaminocyclohexane (DACH) carrier, a modification that imparts unique properties (136). One of the significant advantages of oxaliplatin over its predecessors is its comparatively lower toxicity. This reduction is attributed to its decreased propensity to form DNA-intrastrand cross-links (ICLs) and protein-DNA cross-links (DPCs) (137). While oxaliplatin is transported by the organic cation transporter 2 (OCT2) similar to cisplatin, its nephrotoxicity is partly reduced due to the involvement of multidrug and toxin extrusion transporters (MATEs), which facilitate its excretion and reduce its accumulation in renal tissues (138).

AKI resulting from oxaliplatin administration is rare, with mechanisms that remain incompletely understood. Clinically, AKI may present with oliguria or anuria, and most cases are attributed to ATI, as confirmed by biopsy (4, 139–141). A possible mechanism is direct nephrotoxicity following repeated exposure to the drug. However, there is documentation of AKI occurring after a single dose of oxaliplatin in a patient with pre-existing kidney disease, suggesting that even minimal exposure can precipitate significant renal impairment in this situation (4).

Cases of autoimmune hemolytic anemia (AIHA) progressing rapidly to ATI have been documented, including a fatality. In such scenarios, prompt and aggressive treatment is crucial. Effective interventions include corticosteroid therapy, plasmapheresis, and hemodialysis (142–146). A few documented cases have highlighted histopathologically proven AIN in patients experiencing AKI subsequent to hypersensitivity reactions to oxaliplatin, typically exhibiting allergic symptoms and sterile pyuria preceding kidney failure. Early administration of steroids has demonstrated remarkable efficacy in ameliorating AIN cases (8, 147, 148)..

A patient diagnosed with genuine hemolytic uremic syndrome (HUS) in conjunction with oxaliplatin exposure was successfully managed through a therapeutic regimen comprising IV hydration, loop diuretics, high-dose corticosteroids, and fresh frozen plasma infusions (149). Conversely, two cases initially presenting with clinical manifestations suggestive of TMA were subsequently found, upon comprehensive evaluation including kidney biopsy, to be indicative of AIN and ATI. These instances underscore the pivotal role of a biopsy in accurately diagnosing oxaliplatin-induced AKI (148, 150).

Irrespective of etiology, repeated exposure to oxaliplatin appears to heighten its nephrotoxic potential. Although most cases necessitate hemodialysis and prompt cessation of oxaliplatin, a majority of patients exhibit complete renal recovery. Anemia frequently accompanies oxaliplatin-induced AKI cases, mandating vigilant monitoring of both renal function and hematological parameters throughout oxaliplatin therapy.

### 3.2 Antimetabolites

#### 3.2.1 Methotrexate

Methotrexate (MTX) is an antimetabolite of folic acid, well-regarded for its immunosuppressive and chemotherapeutic properties (151). Approved by the FDA in 1953 for cancer treatment, MTX is among the most extensively employed and investigated anticancer medications. Its mechanism of action entails the inhibition of the enzyme dihydrofolate reductase (DHFR), responsible for catalyzing the conversion of inactive folic acid (dihydrofolate) into its active form, tetrahydrofolate (THF). THF is essential for purine nucleotide synthesis, and by disrupting this pathway, MTX inhibits the replication of cellular DNA, thereby inducing cell death (152).

Nephrotoxicity is associated with high-dose methotrexate (HDMTX), characterized by doses surpassing 500 to 1,000 mg/m2, frequently administered for specific malignancies (153). Renal injury mainly results from MTX and its principal metabolite, 7-OH-methotrexate, forming crystals in the distal renal tubules, consequently leading to ATI (154). Furthermore, a direct tubular toxicity is observed, and might also arise from diminished adenosine deaminase activity induced by oxygen radicals that culminates in tubular necrosis (155, 156). A direct hemodynamic effect has been described, resulting in the constriction of the afferent arterioles or mesangial cells and reducing GFR (10). Consequently, impaired MTX clearance, primarily through renal excretion (over 90%), prolongs exposure to toxic concentrations (157). Certain medications such as non-steroidal anti-inflammatory drugs, salicylates, and antibiotics especially piperacillin-tazobactam and vancomycin, have been described in the literature as exacerbating the risk of AKI when co-administered with MTX (20, 158–161). A genetic mutation in multidrug resistance protein 2 (MRP2) may also confer a higher risk due to impaired MTX elimination (19). Advanced age, male sex, and previous chronic kidney disease (CKD) have also been associated with the development of AKI related to HDMTX treatment (16).

AKI can occur during or post-MTX treatment, with an incidence ranging from 1.8% to 9.1%, despite supportive measures (16, 162). HDMTX induced-AKI is classically present as non-oliguric renal injury and peaks within 1 week. A study carried out in 2010 reported the peak of sCr levels on day 4 post-HDMTX administration, with the recovery of kidney function occurring around day 22. In addition, 65% of patients received diuretics, and 27% underwent hemodialysis with 12 deaths recorded, 6 attributed to irreversible MTX-induced changes. Remarkably, 12 of the 88 survivors received subsequent HDMTX doses after renal function recovery without experiencing toxicity (163).

One of the pillars for the prevention of MTX-related AKI is the alkalinization of the urine. Elevating urine pH enhances the solubility of MTX and its metabolites. Studies indicate that maintaining a urine pH higher than 7.0 can amplify MTX solubility, thereby reducing the formation of intratubular crystals (164). This alkalinization is achieved by administering fluids containing 100-150 mEq/L of sodium bicarbonate at a rate of 125 to 150 mL/h, initiating 12 hours before and continuing for at least 72h after HDMTX treatment until serum MTX concentration is below 0.1–0.2 μmol/L (165, 166). Moreover, monitoring of urine pH every 2 to 4 hours is recommended; if acidity persists, boluses of sodium bicarbonate should be administered to reach the target pH (22). Acetazolamide at a dose of 250-500 mg orally four times daily can serve as an adjuvant for achieving alkaline pH (167, 168)..

Rescue therapy with Leucovorin (Folinic acid) remains a pivotal element in preventing HDMTX toxicity. It should start between 24-48 hours after the beginning of MTX administration, with doses of 25mg or higher given IV every 6 hours, tailored according to MTX concentration, until it is <0.1μmol/L (169). A new strategy involves the use of glucarpidase, a recombinant form of carboxypeptidase-G2 (CPDG2). Glucarpidase hydrolyzes MTX into glutamate and the inactive metabolite DAMPA, which exhibits a quicker elimination rate. This approach is particularly beneficial for patients with delayed MTX excretion or AKI and plasma concentrations of MTX exceeding 1 μmol/L (170). Administering glucarpidase at a dose of 50 U/kg IV over 5 minutes has shown to decrease plasma concentrations of MTX by ≥ 97% or more within 15 minutes (171). CPDG2 does not affect intracellular concentrations of MTX and leucovorin should be administered subsequently based on MTX concentration (163).

High-flux hemodialysis effectively diminishes plasma MTX, however, its effectiveness is curtailed by post-dialysis plasma rebound, and also studies have shown the superiority of CPDG2, with no increases in time of renal function recovery (162).

Recent studies assessing urinary biomarkers for predicting AKI induced by HDMTX have shown that elevated levels of the combination of tissue inhibitor of metalloproteinases-2 and insulin-like growth factor-binding protein 7 (TIMP2∗IGFBP7) is associated with an increased risk of HDMTX-related AKI. However, NGAL has not been demonstrated to be a reliable indicator (172).

#### 3.2.2 Pemetrexed

Pemetrexed, an antifolate agent, serves as a cornerstone in the treatment regimen for non-small cell lung cancer and mesothelioma (173). Its pharmacological efficacy lies in inhibiting key enzymes within the folate metabolic pathway and impeding the synthesis of purine and thymidine nucleotides (174). Renal excretion primarily accounts for its elimination, with a substantial portion of the administered dose recovered in the urine within a 24-hour timeframe (175).

Several case reports have documented instances of acute or subacute kidney injury in patients undergoing pemetrexed therapy, either as a monotherapy or in combination with other chemotherapy drugs. The mechanisms underlying AKI in such cases exhibit considerable variability, with histologically confirmed ATI being the most prevalent, probably occasioned by drug accumulation and interference with folate metabolism. AKI attributed to pemetrexed typically manifests within the initial two weeks of treatment initiation and persists as non-dialytic irreversible renal failure after discontinuation of the drug (176–179).

Additionally, cases of nephrogenic diabetes insipidus and renal tubular acidosis linked with AKI induced by pemetrexed have been reported (5, 180). Clinical trials have revealed a range of nephrotoxicity incidence rates, notably between 7% to 10.7% in patients undergoing maintenance treatment with pemetrexed (181, 182). Retrospective studies have reported higher incidences, ranging from 21% to 30%, with approximately 8% necessitating pemetrexed discontinuation due to AKI development (183, 184).

A significant association between pemetrexed pharmacokinetics and CrCl has been established, wherein higher GFR values before treatment correspond to lower incidences of pemetrexed-induced AKI. Consequently, caution is advised in administering this chemotherapy agent to patients with CrCl below 30-45 ml/min due to heightened renal toxicity risks (175, 176, 185). Moreover, the cumulative dosage, particularly exceeding 10 cycles of pemetrexed-based therapy, raises the likelihood of AKI occurrence (184).

Thymidine may be considered an antidote for pemetrexed-related toxicity if administered within 36 hours of the drug infusion, however, the interpretation of this positive outcome is complicated by the simultaneous use of hemodialysis in the reported case (186).

To mitigate the risks associated with pemetrexed-induced nephrotoxicity, pre-treatment folic acid supplementation is recommended and should be continued throughout therapy to alleviate the detrimental effects on renal function induced by the drug administration (187).

#### 3.2.3 Clofarabine

Clofarabine, a purine nucleotide analog, exerts its pharmacological effects by inhibiting DNA synthesis and ribonucleoside reductase and is primarily excreted by the kidneys (188). It has been used in the treatment of pediatric acute lymphoblastic leukemia and relapsed or refractory adult acute myeloid leukemia (189).

Nephrotoxicity associated with clofarabine has been shown in clinical studies (14, 190–192). The precise mechanisms underlying clofarabine-induced AKI remain unclear. Collapsing glomerulopathy or severe tubular injury, or a combination of both, may be implicated as suggested by animal studies (193). This hypothesis is consistent with a case of biopsy-proven ATI with regenerative nuclear atypia secondary to clofarabine administration (14).

The incidence of AKI associated with clofarabine ranged from 10% to 36% in clinical studies (190, 191). In a cohort involving patients undergoing hematopoietic stem cell transplantation, the incidence of clofarabine-induce AKI was even higher, with 55% of the patients presenting this complication (14).

The onset of AKI has been shown to occur between 6 to 9 days following the initiation of treatment, grade 3 and 4 AKI occurred in 6% to 19% of patients, and a significant of them require temporary hemodialysis (191, 192, 194). Instances of severe AKI were also documented in case reports, including a case showing associated nephrotic-range proteinuria (4.1 g/24h) (193, 195).

Age has been shown as an independent risk factor for clofarabine-induced AKI, suggesting the necessity for dose adjustments in elderly patients even if they present with normal renal function. Higher clofarabine area under the concentration-time curve (AUC) has been associated with more severe AKI cases, suggesting a direct cytotoxic effect on the kidneys (14). Clofarabine-induced AKI occurs more frequently in patients with other risk factors such as baseline CKD, tumor lysis syndrome (TLS), sepsis, and hypotension, as evidenced by multiple studies (191, 192, 194).

In patients who developed clofarabine-induced AKI, the pre-treatment GFR was lower when compared to patients who did not experience this side effect, even if the filtration rate was considered normal at baseline. As the renal injury occurs several days after the drug exposure, medication discontinuation is often inefficient to prevent the injury. Therefore it is imperative to recognize other markers that may indicate a higher susceptibility to the nephrotoxic effect of this drug (14).

The drug’s labeling suggests a 50% dose reduction for patients with a CrCl between 30-60 mL/min, though no specific guidelines are provided for patients with CrCl below 30 mL/min (196).

#### 3.2.4 Gemcitabine

Gemcitabine, a nucleoside analog administered intravenously, received approval from the FDA in 1996. Initially sanctioned as a monotherapy for pancreatic cancer, its utilization has expanded to combination therapy with other chemotherapeutic agents for the treatment of various solid organ malignancies (197).

Despite its therapeutic efficacy, the administration of gemcitabine is associated with a 2.9% incidence of AKI (1). Additionally, gemcitabine-induced TMA is well recognized, presenting as a syndrome encompassing different degrees of microangiopathic hemolytic anemia, thrombocytopenia, neurologic, and renal dysfunction (198).

A study involving 120 cases of TMA associated with gemcitabine revealed that 97.4% of affected individuals developed AKI, with 27.8% necessitating hemodialysis (199). Histopathological examination of kidneys afflicted by acute TMA reveals thrombi or fragmented red cells in the mesangial space, mesangiolysis, endothelial cell swelling, and luminal occlusion by thrombi (200). The precise mechanism of TMA onset remains incompletely elucidated; however, several hypotheses have been posited. One theory suggests that direct endothelial injury may be the primary inciting event, while others speculate about the generation of antibodies against ADAMTS13 as a trigger for the syndrome (7). The primary risk factor associated with the development of TMA is a cumulative dose exceeding 20,000 mg/m² (7). New or worsening hypertension can be an early indicator of TMA in patients receiving gemcitabine treatment (201).

The discontinuation of therapy is the initial approach for patients diagnosed with gemcitabine-induced nephrotoxicity. Although plasmapheresis has been explored, its efficacy remains limited. Rituximab and eculizumab have demonstrated potential benefits in some cases (202–204).

Patients exhibiting altered renal function were specifically excluded from phase I trials involving gemcitabine, thereby precluding the establishment of any recommended dosage for this particular population. Nonetheless, a study suggests that patients with ESRD undergoing hemodialysis may benefit from gemcitabine treatment without dose reduction. This phenomenon is likely attributable to the effective elimination of the inactive metabolite 2’,2’-difluorodeoxyuridine (dFdU) via dialysis, starting 6-12 hours post-chemotherapy administration, thereby potentially safeguarding the patient without compromising the cytotoxicity against tumors. Vigilant monitoring of treatment tolerance is crucial to sustaining efficacy while adjusting doses and considering the concurrent use of other nephrotoxic agents. Furthermore, continuous monitoring for three months following the completion of treatment is strongly recommended (205, 206).

#### 3.2.5 Capecitabine

Capecitabine, an FDA-approved pro-drug since 1998, is employed in the treatment of various solid malignancies, including breast and colorectal cancers (207–209). Dihydropyrimidine dehydrogenase (DPD) is the primary enzyme responsible for the metabolism and clearance of over 85% of 5-fluorouracil (5-FU), the active metabolite of capecitabine (210). A genetic deficiency in DPD significantly increases the risk of severe toxicity following capecitabine administration due to the accumulation of 5-FU in the body (211, 212).

AKI induced by capecitabine has been documented in clinical studies, with incidences ranging from 8.3% to 17%, depending on treatment protocols and the use of combination chemotherapeutic agents (213, 214). The exact mechanism underlying capecitabine-induced AKI remains elusive. However, a compelling theory suggests that reversible vasoconstriction of vascular smooth muscle, triggered by the activation of protein kinase C by 5-FU, leads to preglomerular vasoconstriction, reduced glomerular filtration, and subsequent renal failure. This hypothesis is supported by observations of cardiovascular toxicity induced by 5-FU, specifically coronary vasospasm leading to acute myocardial ischemia and myocardial infarction (215, 216).

A recent case report documented TMA as a possible underlying cause of renal failure induced by capecitabine. In this case, the diagnosis of TMA secondary to capecitabine was confirmed through renal biopsy in an 82-year-old woman with localized colon adenocarcinoma, who had no prior history of renal disease. Following the discontinuation of capecitabine, the patient showed significant improvement in renal function (6).

Furthermore, renography has emerged as a sensitive early diagnostic tool for detecting declines in renal function during capecitabine treatment, potentially used even for AKI induced by other chemotherapeutic agents. This approach was highlighted in a case report where a patient experienced AKI during capecitabine treatment, early evidenced by renography, which possibly prompted discontinuation of the drug and total reversibility of renal failure (217).

Research indicates that with rigorous monitoring and appropriate dose reductions (50-80%), capecitabine can be safely administered to patients with severe and ESRD, allowing these patients to benefit from its antitumor efficacy without further compromising renal function (218).

In attempting to mitigate kidney and hepatic damage induced by capecitabine, experimental studies in rats have shown that caffeic acid administration can be effective. Caffeic acid enhances the antioxidant defense system and reduces lipid peroxidation, however further studies in humans are needed to confirm these findings (219).

#### 3.2.6 Cytarabine

Cytarabine, also known as Cytosine Arabinoside (Ara-C), is a pyrimidine nucleoside analog utilized in the treatment of leukemias and lymphomas (220). Its primary mechanism of action involves the inhibition of DNA polymerase, thereby halting DNA replication and repair (221). Myelosuppression is the most significant adverse effect of cytarabine, occurring even at low doses (222). Additionally, cytarabine poses a risk of inducing AKI, with an incidence rate of 4.8% (1).

Cytarabine is implicated in TLS, an important factor in the onset of AKI due to the deposition of urate and calcium phosphate in the renal tubules (223).

In a study comparing treatment protocols, 85% of patients on the Ara-C-DaunorubicinAra-C protocol exhibited impaired renal function, compared to 55% of those receiving Ara-C combined with cyclophosphamide. The diagnosis of AKI in these patients was based on the elevation of sCr to twice normal and/or a reduction in CrCl to less than 50% of the baseline value. Autopsies of affected patients revealed interstitial edema, tubular dilatation, epithelial flattening, focal atypia, and occasional mitotic figures in the tubular epithelium (224).

As the metabolite of Ara-C is typically excreted in the urine, patients with renal impairment experience reduced renal clearance of the drug, thereby increasing the risk of severe toxicity. Despite these findings, the FDA does not recommend adjusting the cytarabine dosage for patients with compromised kidney function (225). One potential preventive measure investigated was the co-administration of low-dose dopamine with cytarabine; however, this combination did not demonstrate a significant reduction in nephrotoxicity (226).

### 3.3 Antitumor antibiotics

#### 3.3.1 Mitomycin C

Mitomycin C is an antibiotic introduced as an anticancer agent in 1958, primarily recognized for its efficacy in treating breast and gastrointestinal cancers by acting through DNA alkylation (227).

AKI can be a fatal complication of this drug and usually occurs in the context of TMA. Hemolytic Uremic Syndrome (HUS) affects 4% to 5% of patients treated with mitomycin C. This complication is dose-dependent and usually occurs in patients treated with doses above 60 mg/m2, rarely seen in doses of less than 30mg/m2 (228). A latency period of 2 to 5 months has been reported between the dose of mitomycin C and the onset of HUS (229). Preventive measures include drug discontinuation as soon as the syndrome is suspected and supportive care (230). Renin-angiotensin system inhibitors can be used to reduce systemic glomerular hypertension and high-grade proteinuria, when necessary (200). Supporting this assertion, there is a case report showing a possible improvement in diuresis and renal function after treatment with captopril, indicating that studies on the use of this medication in patients with mitomycin C-induced HUS need more attention (229). There have also been reports of successful remission of severe cases of TMA associated with mitomycin C using protein A immunoadsorption, considering the hypothesis that immune complexes are involved in this pathogenesis (23). Conversely, Rituximab was tested but did not yield positive outcomes. Additionally, platelet transfusion should be avoided in microangiopathic thrombosis in the absence of bleeding, as it is associated with a worsening of microvascular thrombosis. In more severe cases of kidney disease, dialysis therapy should be considered (200).

Dose adjustment is discouraged for patients with a history of renal dysfunction due to mitomycin C’s poor renal excretion. In cases of severe pre-existing renal disease, it is advisable to consider non-nephrotoxic alternatives whenever feasible for these patients (231).

#### 3.3.2 Doxorubicin

Doxorubicin (DOX) is an anthracycline agent extensively used to treat malignancies, including hematologic and breast cancers. Its antitumor activity is primarily attributed to its ability to inhibit the synthesis of DNA, RNA, and proteins, resulting in cellular death (232).

Among anthracycline medications approved by the FDA, DOX stands out as both the most potent agent and the one most commonly associated with nephrotoxicity, constituting a major dose-limiting adverse effect (233). Although the precise mechanism underlying DOX-induced kidney damage remains incompletely understood, research indicates that it predominantly involves podocyte injury secondary to oxygen-free radicals and a tubulointerstitial inflammatory response (234). DOX disrupts mitochondrial function, generating superoxides and depleting antioxidant compounds (233).

The most prevalent renal toxicity associated with DOX is severe proteinuria, which can progress to focal glomerulosclerosis when protein loss is sustained. Furthermore, the development of structural glomerular damage is associated with hypertension, steroid resistance, and a high incidence of progression to renal failure (232, 233). Some strategies have been explored to mitigate DOX’s nephrotoxic effects, including the administration of antioxidant compounds, modifications to the drug delivery system, and the development of analogues; however, these approaches have not yielded significant clinical success (232).

An alternative strategy introduced in the 1990s involved the use of Pegylated Liposomal DOX (L-DOX) which aims to reduce cardiac absorption and extend the drug’s half-life. While this formulation has been effective in diminishing cardiac toxicity, it has not demonstrated similar efficacy in reducing renal injury, occurring even months post discontinuation of L-DOX, and is commonly non-reversible (235). Prolonged exposure to L-DOX in a patient with metastatic breast cancer led to AKI and progressively worsening kidney failure, necessitating dialysis and persisting long after the cessation of treatment (236).

Fasudil, a Rho/ROCK inhibitor, demonstrates anti-inflammatory, antioxidant, and anti-apoptotic properties and exhibits promising potential in mitigating DOX-induced nephrotoxicity, as evidenced by animal and cell culture studies; however, further evaluation in clinical studies is warranted to ascertain its safety and effectiveness in humans (237).

### 3.4 Alkylating agents

Alkylating agents are a class of drugs that inhibit DNA transcription by cross-linking DNA strands, thereby preventing nucleic acid replication and inducing apoptosis. They are categorized into six principal types: nitrogen mustards (e.g., mechlorethamine, cyclophosphamide, ifosfamide, melphalan, chlorambucil), ethylenamine and methylamine derivatives, alkylsulfonates, nitrosoureas, triazines, and platinum-containing antineoplastic agents. Some of these agents will be reviewed here as they are known to cause AKI.

#### 3.4.1 Ifosfamide

Ifosfamide (IFO) is a chemotherapeutic agent analogous to cyclophosphamide, employed in the treatment of various solid tumors, including sarcomas. However, a significant adverse effect associated with IFO is nephrotoxicity, primarily attributed to its principal metabolite, chloroacetaldehyde. This metabolite induces proximal tubular dysfunction (PTD), the most common form of renal damage observed with IFO use (238).

IFO-induced AKI extrapolates this mechanism and occurs through a dose-dependent inhibition of renal thioredoxin reductase activity, as evidenced by animal models. This inhibition disrupts both the thioredoxin and GSH systems, particularly in patients with reduced renal GSH levels, thereby increasing the risk of AKI induced by IFO (239).

The majority of studies center around pediatric populations. A cohort study involving 34 adult patients provided notable insights. Among these individuals, 19 patients were exclusively treated with IFO, resulting in 10 cases experiencing AKI alongside PTD, with isolated AKI observed in only one case. In contrast, when cisplatin was concurrently administered with IFO, it correlated with a higher incidence of isolated AKI (238). The suggestion of augmented nephrotoxicity risk with simultaneous or prior cisplatin administration is supported by additional studies, indicating that the decline in renal function is modest unless IFO is used with cisplatin (240). Furthermore, the risk of renal injury escalates with cumulative doses, although the exact threshold varies. AKI can manifest even at cumulative doses below 60 g/m2, influenced by factors such as age and genetic predisposition (238).

Currently, there are no established therapies for the prevention or treatment of IFO-induced AKI. However, preclinical studies suggest that NAC may offer renal protection during IFO therapy while maintaining its antitumor efficacy. NAC’s protective mechanism is likely through its role as a precursor for GSH synthesis, with efficacy dependent on its concentration (241, 242). In contrast, corticosteroids have shown limited efficacy, with patients often progressing to CKD despite treatment (238).

In clinical practice, it is recommended to promptly discontinue IFO upon the identification of kidney failure to mitigate IFO-induced AKI (238). Adjusting the dosage of IFO may also be necessary for patients with pre-existing renal disease. However, prospectively validated dosing guidelines, including the use of clinical decision support systems, may be needed for consistent dose adjustment in cases of renal impairment (243).

#### 3.4.2 Trabectedin

Trabectedin represents a crucial therapeutic avenue for patients confronting limitations in tolerating conventional chemotherapy agents, notably IFO or DOX, either due to intolerable side effects or following unsuccessful initial chemotherapy regimens. Its principal adverse effect is hepatotoxicity, with sporadic occurrences of rhabdomyolysis and renal toxicity documented in the literature. Although the precise mechanism of renal injury remains unclear, there is a possible link between elevated creatine phosphokinase (CPK) levels and AKI, indicating potential myoglobin-induced damage (244, 245).

Clinical trials on trabectedin have reported a low incidence of rhabdomyolysis with AKI (0.7%) and isolated CPK elevation levels without concurrent AKI (0.4%). Among cases of AKI, a considerable proportion necessitated hemodialysis (26%), while renal recovery was achieved in a third of cases (33%), with a substantial portion resulting in mortality (41%) (245, 246).

Additionally, severe AKI and mortality associated with trabectedin have been documented even in the absence of rhabdomyolysis (247).

In clinical practice, it is recommended to promptly discontinue trabectedin upon the diagnosis of rhabdomyolysis, as delaying cessation and initiation of management could potentially exacerbate trabectedin-induced AKI and precipitate fatal outcomes (245). According to the drug’s label, if a patient’s creatinine phosphokinase (CPK) levels exceed 2.5 times the upper limit of normal or if they experience any other non-hematologic adverse reactions graded 3 or 4, dosing should be delayed for up to 3 weeks. If CPK levels are greater than 5 times the upper limit of normal, the subsequent dose of trabectedin should be reduced by 1 dose level. Dose adjustment is not recommended in patients with mild or moderate renal impairment (248).

#### 3.4.3 Bendamustine

Bendamustine has exhibited notable efficacy in the clinical management of hematologic malignancies, particularly in scenarios involving indolent non-Hodgkin lymphoma and chronic lymphocytic leukemia, where patients have either relapsed or exhibited resistance to previous treatment modalities. This efficacy can be attributed to its dual mechanism of action, encompassing both alkylating agent properties and antimetabolite activity (249).

In a retrospective analysis of 122 patients undergoing intensified bendamustine therapy, a significant portion (41.8%) experienced AKI, and the majority of cases were reversible, with a reversal rate of 98%. The manifestation presented with an average duration of 7 days, ranging from 1 to 22 days, and the necessity for transient hemodialysis was minimal, occurring in only three cases. Factors correlating with the incidence of bendamustine induced-AKI include advanced age, typically exceeding 60 years, during autologous stem cell transplantation. Previous occurrences of AKI and pre-existing kidney disease preceding high-dose chemotherapy also confer an augmented risk. Additionally, the concurrent administration of nephrotoxic agents exhibited a predisposition toward heightened AKI rates during bendamustine treatment, while concomitantly increasing the likelihood of cardiovascular complications among affected patients (17).

In a comparative investigation delineating conditioning chemotherapy regimens for non-Hodgkin lymphoma, the use of bendamustine as a substitute for carmustine in combination with etoposide, cytarabine, and melphalan (the BE-EAM regimen) was associated with a significantly elevated incidence of nephrotoxicity compared to the BEAM regimen, in which only 7% of patients exhibited renal impairment. Specifically, 31% of patients in the BE-EAM cohort developed early renal toxicity before the day of reinfusion, and, after reinfusion, the cumulative incidence of nephrotoxicity had risen to 48%, with 17% of cases being grade 3–4. The hallmark features of AKI in this context were characterized by an early surge in Scr levels after chemotherapy initiation, accompanied by suggestive findings indicative of tubular impairment upon urinary ionogram analysis. However, renal biopsy confirmation was not performed. Notably, all patients exhibited complete restoration of renal function within a three-week timeframe, supporting that Bendamustine-induced AKI is predominantly reversible (249).

#### 3.4.4 Streptozocin

Streptozotocin (STZ) is a chemotherapeutic agent widely employed in the treatment of neuroendocrine tumors (250). Its use has been associated with nephrotoxicity, in which the mechanism possibly involves DNA damage and the subsequent activation of p53 in tubular epithelial cells, resulting in proximal tubular injury (251). The renal damage often manifests initially with hypophosphatemia, glycosuria, and proteinuria preceding the decline in renal function, serving as an early biomarker of renal injury (252).

In a study involving 52 patients treated with STZ for pancreatic islet cell carcinoma, renal toxicity affected 65% of the cohort. Although AKI was not the predominant renal manifestation, occurring in 26% of cases, it proved to be the most fatal adverse event, resulting in 11% mortality. Other renal manifestations included proteinuria (51%), renal tubular acidosis (17%), and Fanconi syndrome (13%) (250).

Additionally, a case report underscores a potential delayed onset of nephrotoxicity linked to STZ administration. It details a patient who displayed only mild renal impairment during and shortly after discontinuing STZ treatment. Intriguingly, CKD manifested in this individual a year after the cessation of STZ therapy, shedding light on the possibility of delayed renal complications associated with STZ use (252).

A potential preventive measure to mitigate STZ-induced AKI targeting the mechanism of tubular injury associated with this chemotherapeutic drug has been investigated. STZ gains cellular entry through GLUT2 and SGLT2 transporters, primarily inducing tubular injury in the outer cortex, where these transport proteins are highly expressed. *In vivo* studies have demonstrated that SGLT2 inhibitors hold promise in averting DNA damage in tubular epithelial cells, thereby mitigating renal injury. This protective effect on renal function was achieved while preserving the antitumor efficacy of STZ, suggesting that pretreatment with an SGLT2 inhibitor is a preventive option (251).

A relative contraindication for STZ administration is pre-existing renal disease. If employed in such patients, dosage adjustments should be individualized based on clinical status with close monitoring (250).

#### 3.4.5 Melphalan

Melphalan is a chemotherapeutic agent commonly used in the treatment of multiple myeloma. Its renal excretion is minimal, ranging from 5.8% to 21.3%, contributing to the idea that this drug could be non-nephrotoxic. However, evidence suggests that certain patients may experience kidney injury, albeit rarely (21).

A study involving patients with AL amyloidosis and pre-existing tubular injury who received high-dose melphalan revealed the development of ATI. The study indicates that pre-existing tubular dysfunction might be a prerequisite for renal injury. Additionally, oval fat bodies were detected in 60% of patients who developed AKI after high-dose melphalan, compared to only 25% of those without AKI, leading to the conclusion that it could serve as a predictor of AKI following high-dose melphalan administration (21).

The incidence of AKI in patients receiving melphalan ranges from 1.6% to 18.8% and its occurrence has been linked with poorer survival rates (21, 253). Several risk factors contribute to the development of AKI, including advanced age, high urine sediment score, hypoalbuminemia, nephrotic-range proteinuria, and diuretic usage. Among these factors, age and urine sediment score emerged as the most significant predictors. Patients aged 50 years or older with high urine sediment were found to be 10 times more likely to develop AKI compared to those lacking these risk factors (with an incidence of 73% versus 7%) (21).

While a study conducted on rats suggested a potential protective effect of quercetin against melphalan-associated AKI, there is currently no available data regarding its efficacy in humans (254).

The literature presents varying recommendations for dose adjustment of melphalan. According to the FDA, in the palliative setting, patients with pre-existing renal insufficiency with BUN levels ≥ 30 mg/dl could undergo a 50% dose reduction due to the heightened risk of myelosuppression. However, in the conditioning treatment, all patients should receive the full dose (253). In contrast, the International Myeloma Working Group offers different recommendations based on the patient’s CrCl. For patients with normal renal function, the oral melphalan dose should range from 0.15 to 0.25 mg/kg/day. If CrCl is between 15 and 59 mL/min, the melphalan dosage must be reduced by 25%. In cases where CrCl falls below 15 mL/min or if the patient is on dialysis, the oral melphalan dose should be reduced by 50%. For the high-dose IV melphalan protocol, the recommended dose is 200 mg/m2 for patients with normal renal function. However, if CrCl is less than 60 or if the patient is on dialysis, the dose should be reduced to 100-140 mg/m2 (255).

## 4 Conclusion

Chemotherapy-induced AKI presents a significant challenge at the intersection of oncology and nephrology, complicating cancer treatment, particularly with platinum compounds. The overarching objective remains the optimization of renal health in oncologic patients while preserving the antitumor efficacy of their treatment. Understanding the diverse mechanisms of AKI is essential and requires further elucidation, as they vary among the different classes of chemotherapeutic agents. Tailoring precise protective measures, in addition to general strategies such as hydration protocols, is crucial in mitigating this limiting side effect. Further clarification of risk factors is needed, and early diagnosis is essential for initiating therapeutic interventions, including dose adjustments and drug discontinuation. Addressing knowledge gaps, overcoming challenges, and incorporating emerging trends in prevention and management are essential to reduce the burden associated with AKI induced by conventional chemotherapy.
